# Early treatment of neonatal diabetes with oral glibenclamide in an extremely preterm infant

**DOI:** 10.1002/jmd2.12358

**Published:** 2023-01-29

**Authors:** Alfonso Galderisi, Elsa Kermorvant‐Duchemin, Alejandra Daruich, Adeline Alice Bonnard, Alexandre Lapillonne, Marie‐Stéphanie Aubelle, Bruna Perrella, Yoann Vial, Héléne Cave, Marianne Berdugo, Pierre‐Henri Jarreau, Michel Polak, Jacques Beltrand

**Affiliations:** ^1^ Hôpital Universitaire Necker‐Enfants Malades, Service d'endocrinologie Gynécologie et Diabétologie Pédiatrique Hôpital Necker‐Enfants Malades Paris France; ^2^ Department of Woman and Child's Health University of Padova Padova Italy; ^3^ Department of Neonatal Medicine Hôpital Universitaire ‐ Enfants Malades, Université Paris Cité Paris France; ^4^ Inserm, Centre de Recherche des Cordeliers, Sorbonne University Paris Cité University, Physiopathology of Ocular Diseases: Therapeutic Innovations Paris France; ^5^ Ophthalmology Department Necker‐Enfants Malades University Hospital, Assistance Publique Hôpitaux de Paris, Paris Cité University Paris France; ^6^ Département de Génétique Hôpital Universitaire Robert Debré Paris France; ^7^ INSERM UMR_S1131 ‐ Institut de Recherche Saint‐Louis Paris France; ^8^ Hôpital Universitaire Necker‐Enfants Malades, Service de Pédiatrie et Réanimation Néonatales Université Paris Cité Paris France; ^9^ Neonatal Intensive Care Unit of Port‐Royal APHP. Centre ‐ Université Paris Cité, APHP Paris France; ^10^ Institut IMAGINE, INSERM U1163 Paris France; ^11^ Institut Cochin INSERM U1016 Paris France; ^12^ Centre des maladies endocriniennes rares de la croissance et du développement Hôpital universitaire Necker‐Enfants malades Paris France

**Keywords:** glibenclamide, monogenic diabetes, neonatal diabetes, prematurity

## Abstract

Early treatment of neonatal diabetes with sulfonylureas has been proven to produce marked improvements of neurodevelopment, beside the demonstrated efficacy on glycemic control. Several barriers still prevent an early treatment in preterm babies including the limited availability of suitable galenic form of glibenclamide. We adopted oral glibenclamide suspension (Amglidia) for the early treatment of neonatal diabetes due to an homozygous variant of KCNJ11 gene c.10C>T [p.Arg4Cys] in an extremely preterm infant born at 26 + 2 weeks' of gestational age. After ~6 weeks of insulin treatment with a low glucose intake (4.5 g/kg/day), the infant was switched to Amglidia 6 mg/ml diluted in maternal milk, via nasogastric tube (0.2 mg/kg/day) progressively reduced to 0.01 mg/kg/day (after ~3 months). While on glibenclamide, the patient exhibited a mean daily growth of 11 g/kg/day. The treatment was suspended at month 6 of birth (weight 4.9 kg [5th–10th centile], M3 of c.a.) for normalization of glucose profile. During the treatment, the patient exhibited a stable glucose profile within the range of 4–8 mmol/L in the absence of hypo or hyperglycemic episodes with 2–3 blood glucose tests per day. The patient was diagnosed with retinopathy of prematurity Stade II in Zone II without plus disease at 32 weeks, with progressive regression and complete retinal vascularization at 6 months of birth. Amglidia could be regarded as the specific treatment for neonatal diabetes even in preterm babies due to its beneficial effect on the metabolic and neurodevelopmental side.

**Synopsis**

Glibenclamide is effective for the treatment of transient neonatal diabetes in an extremely preterm infant.

The use of low‐dose neonatal formualtion (Amglidia 06.mg/ml) may facilitate dose titratrion).

## 1 INTRODUCTION

The use of sulphonylureas for the treatment of neonatal diabetes secondary to mutations in potassium‐channel subunits has been proven to produce marked improvements in neuropsychomotor development,^1^ including the domains of attention, motor coordination, and language. Such an effect, besides the largely demonstrated metabolic benefits,^2^, ^3^ seems to be inversely correlated with the age‐of‐treatment onset, with earlier treatment showing more pronounced improvement in visual and motor neurodevelopmental domains.^4^, ^5^

Several barriers still prevent an early beginning of treatment in newborns with suspected neonatal diabetes, including the delay of genetic testing and the absence of neonatal‐adapted formulation able to easily titrate neonatal dose and to be adjusted to specific neonatal administration as the need for mixing with maternal or formula milk.

The recent availability of two oral formulation of glibenclamide—Amglidia 6 mg/ml and Amglidia 0.6 mg/ml—approved by the European Medical Agency—has paved the way to an early treatment for neonatal diabetes even in preterm infants thanks to the ease of dose titration and the predictable pharmacokinetics.^6^

Besides the undoubted benefits on glycemic profile, the use of sulphonylureas in preterm infants with hyperglycemia may—in turn—present additional advantages with respect to their potential favorable nonglycemic effects, including brain and retinal neuroprotection described in the murine and nonhuman primates model.^7^

We report on the first use of the oral formulation of glibenclamide (Amglidia) in an extremely preterm infant diagnosed with neonatal diabetes at 26 weeks and started on oral glibenclamide at 32 weeks post‐menstrual age (PMA).

## 2 METHODS

### 2.1 Perinatal clinical characteristics

We report on a female neonate, born at 26 + 2 weeks gestation (birthweight 750 g [10th centile], length 32 cm [40th centile], head circumference 24 cm [50th centile]). She was born after a C‐section for chorioamnionitis, intubated at 30 min of birth for increasing O_2_‐requirements and received a single dose of surfactant 200 mg/kg. After 6 h, she was extubated and started on noninvasive nasal continuous positive airway pressure (nCPAP) that was maintained for 43 days. The post‐natal course was featured by an infection with Candida non‐Albicans (positive meconium) in the absence of a positive hemoculture.

Parenteral nutrition was continued for the first 7 days of birth with progressive weaning and switch to oral nutrition. She was diagnosed with retinopathy of prematurity (ROP) Stade II in Zone II without plus disease, at 32 weeks PMA.

### 2.2 Neonatal metabolic profile

For the detection of hyperglycemia (12.5 mmol/L) on Day 1 of birth, and suppressed C‐peptide (<0.2 nmol/L), insulin treatment was started at the dose of 0.12 U/kg/h with a reduction of glucose intakes at 4.5 g/kg/day. Insulin treatment was maintained until day 41 after progressive weaning with normalization of the glucose profile and increase of daily glucose intakes. The daily growth during insulin treatment was 26 g/kg/day. Before and during the insulin treatment, we never observed neither acidosis nor ketosis (beta‐hydroxybutyrate <1 mmol/L).

**FIGURE 1:**
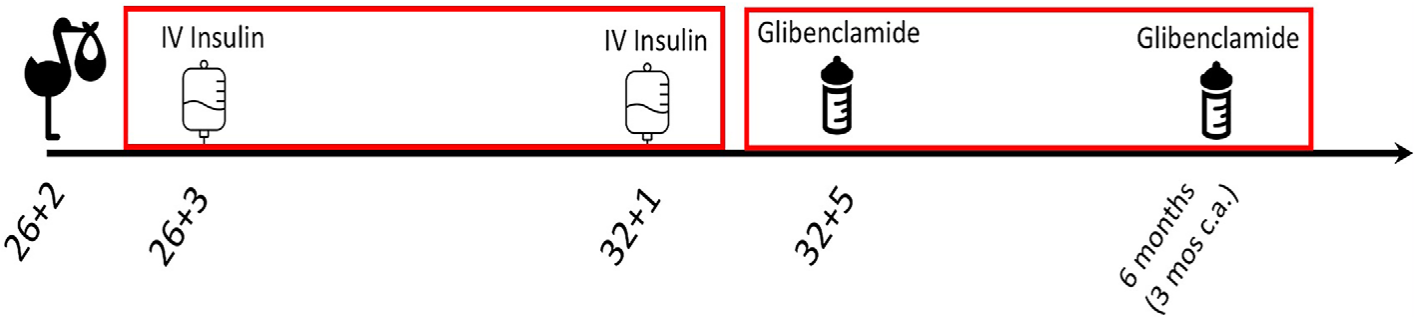
Timeline of treatment

Four days after the suspension of the insulin treatment (D45, age 32 + 5 weeks) we observed a new rise of blood glucose (9 mmol/L) in the absence of ketosis. She was started on glibenclamide at the dose of 0.2 mg/kg/day in two daily administrations using the oral formulation of glibenclamide (Amglidia 6 mg/ml) with normalization of glucose intake (between 6 and 8 g/kg/day). The dose was progressively reduced to 0.02 mg/kg/day over a lag‐time of 12 weeks with normalization of glucose profile (Figure 1). At discharge, she was switched to the 0.6 mg/ml formulation of Amglidia with a daily dose of 0.016 mg/kg/day.

### 2.3 Oral formulation of Amglidia

The two approved oral formulations of Amglidia include, besides glibenclamide, sodium benzoate (as a preservative), a lactic acid/sodium citrate mixture to give the solution a flavorless slight acid pH (= 4.80) that ensures the efficacy of sodium benzoate as preservative, and hydroxyethyl cellulose, and xanthan to prevent sedimentation of the active glibenclamide. Such a formulation has an osmolarity of 282 mOsm/L, close to isotonicity, and can be administered either as a standalone suspension or diluted in milk.^6^

### 2.4 Genetic diagnosis of neonatal diabetes

Aberrant methylation or copy number alteration of the *PLAGL1* gene on chromosomal region 6q24 was ruled out using multiplex methylation‐specific ligation‐dependent amplification (SALSA MS‐MLPA ME‐033 TNDM; MRC‐Holland). NGS analysis of a panel of 24 genes known to be involved in neonatal diabetes evidenced a homozygous variant NM_000525.4:c.10C>T p.(Arg4Cys) in the *KCNJ11* gene, inherited from each asymptomatic heterozygous parent.

This variant was considered likely pathogenic because it is rare in population databases (GnomAD v2.1.1, 6/279042) and has never been observed in the homozygous state (PM2 according to American College of Medical Genetics and Genomics criteria^8^). In silico test predicted a deleterious effect on the protein (PP3^8^). Finally, this variant has been previously reported as pathogenic in patients with transient or permanent neonatal diabetes.^9^

## 3 RESULTS

### 3.1 Glucose management with Amglidia

**FIGURE 2:**
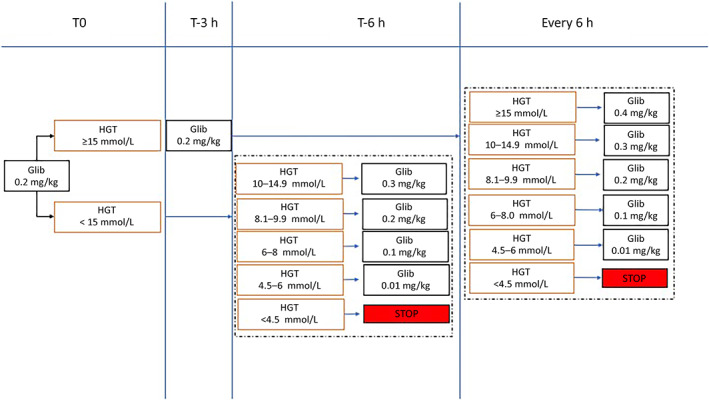
Glibenclamide dose titration protocol. To convert glucose (mmol/L) to glucose (mg/dl) multiply by 18 (e.g., 1 mmol/L = 18 mg/dl).

Amglidia oral solution was administered via nasogastric tube after dilution with maternal milk (0.5 ml of Amglidia 6 mg/ml in 2.5 ml of maternal milk with a final concentration of 1 mg/ml). The starting dose of 0.2 mg/kg/day was titrated according to the protocol displayed in Figure 2. Briefly, glucose was monitored with heel stick determinations every 6 h.

The patient was discharged with Amglidia 0.6 mg/ml at a fixed dose of 0.015 mg/kg/day, less than one‐tenth of the starting dose.

The treatment with glibenclamide was maintained until 6 months of life with a progressive weaning of the dose during month 6. During the treatment, the patient exhibited a mean daily growth of 11 g/kg/day.

### 3.2 Follow‐up assessment

At the 6 month follow‐up visit (weight 4.9 kg [5th–10th centile, −1.1SD] at 3 months, c.a.), the patient exhibited a stable glucose profile within the range of 4–8 mmol/L in the absence of hypo or hyperglycemic episodes with 2–3 glucose tests per day.

The abdominal ultrasound did not display any anatomic abnormality of the pancreas and excluded hepatic and renal cysts.

The clinical neurological assessments conducted at 34 weeks PMA, after 6 and 12 months demonstrated the achievement of the expected milestones according to the corrected age. Retinal fundus examination at 6 months displayed complete peripheral retinal vascularization, without ROP.

Our case demonstrates the safety and efficacy of Amglidia 6 mg/ml and 0.6 mg/ml in a neonate born extremely preterm with treatment started at 32 weeks of age.

## 4 CONCLUSION

The main highlights of the case are the ability of the pediatric formulation to allow a dose titration adequate for this age, the possibility of administering the dose via nasogastric tube at first, and then orally at home. The use of a low‐dose formulation (0.6 mg/ml) allows a customizable therapeutic regimen and prevents prolonged hypoglycemia, a frequent complications of early treatment. In addition, glibenclamide early treatment ensured an adequate growth for the age, as this latter is a key therapeutic goal of early treatment of neonatal diabetes.

Neonatal hyperglycemia is observed in more than one out of four very low birthweight infants^10^, ^11^, ^12^ and generally treated with insulin, even though the actual benefits of insulin treatment remain highly debated.^13^, ^14^ The benefits of sulfonylureas on neurodevelopmental outcome^3^, ^5^ and the unknown actual prevalence of potassium channel mutations in infants developing transient hyperglycemia suggest the need for further investigations aimed to explore the frequency of the mutations in neonatal hyperglycemia as well as the use of glibenclamide as first‐line treatment for neonatal hyperglycemia.

A major limitation of this study remains the absence of continuous glucose monitoring during the treatment that would have supported therapeutic choices as well as documented episodes of hypo and hyperglycemia that might be undetected with point‐of‐care measures of glucose.^15^, ^16^ The causative nature of the observed mutation is very likely, as described. The effect of the early glibenclamide treatment on glycemic control as well as on the weight progression, along with the absence of ketosis at the onset supports the hypothesis of transient neonatal diabetes due to a potassium channel mutation.

The regression of ROP cannot be conclusive for a direct effect of glibenclamide on retinal vascular development since most ROP regress without treatment.^17^ However, hyperglycemia is one of the major risk factors for ROP as shown in two cohorts of preterm infants^18^ and glibenclamide has shown neuroprotective retinal effects.^7^ Further investigation should be granted to determine the role of glibenclamide in ROP physiopathology.

This experience demonstrates the feasibility of oral glibenclamide treatment for hyperglycemia in an extremely preterm infant and suggests the need for wider exploration of this new oral formulation in comparison with the burden and the risk associated with insulin treatment, in the frame of neonatal hyperglycemia of preterm infants.

## AUTHOR CONTRIBUTIONS

Alfonso Galderisi, Jacques Beltrand, and Michel Polak drafted the manuscript and conceptualize the clinical case, and revised the manuscript; Elsa Kermorvant‐Duchemin, Alexandre Lapillonne, Marie‐Stéphanie Aubelle, Bruna Perrella, and Pierre‐Henri Jarreau followed the early and late neonatal care of the infant, then performed the neurodevelopment assessment at follow‐up visit and revised the manuscript. Alejandra Daruich evaluated the retinopathy progression; Adeline Alice Bonnard, Yoann Vial, and Héléne Cave performed the research for genetic mutations associated with the observed metabolic phenotype and revised the manuscript; Michel Polak, Jacques Beltrand, and Marianne Berdugo contributed to the development of the oral suspension of the medication and critically revised the manuscript. Michel Polak is the guarantor of data integrity. All authors approved the final manuscript as submitted and agree to be accountable for all aspects of the work.

## CONFLICT OF INTEREST

Michel Polak has been the scientific advisor for the AMGLIDIA development, none for the others. The other authors declare that they have no conflict of interest.

## FUNDING INFORMATION

This study was funded by ANR 22‐CE17‐0025 Neurogli (to MP).

## ETHICS STATEMENT

Institutional authorizaition to retrospectively access the clinical data was obtained.

## INFORMED CONSENT

This article does not contain any studies with human or animal subjects performed by the any of the authors.

## Data Availability

Data will be made available on reasonable request to the Authors.

## References

[1] Beltrand J , Elie C , Busiah K , et al. Sulfonylurea therapy benefits neurological and psychomotor functions in patients with neonatal diabetes owing to potassium channel mutations. Diabetes Care. 2015;38(11):2033‐2041. 26438614 10.2337/dc15-0837

[2] Pearson ER , Flechtner I , Njølstad PR , et al. Switching from insulin to oral sulfonylureas in patients with diabetes due to Kir6.2 mutations. N Engl J Med. 2006;355(5):467‐477. 16885550 10.1056/NEJMoa061759

[3] Bowman P , Mathews F , Barbetti F , et al. Long‐term follow‐up of glycemic and neurological outcomes in an international series of patients with sulfonylurea‐treated ABCC8 permanent neonatal diabetes. Diabetes Care. 2021;44(1):35‐42. 33184150 10.2337/dc20-1520 PMC7783935

[4] Sha hR , Spruyt K , Kragie B , Greeley S , Msall M . Visuomotor performance in KCNJ11‐related neonatal diabetes is impaired in children with DEND‐associated mutations and may be improved by early treatment with sulfonylureas. Diabetes Care. 2012;35(10):2086‐2088. 22855734 10.2337/dc11-2225 PMC3447845

[5] de Gouvela Buff Passone C , Giani E , Vaivre‐Douret L , et al. Sulfonylurea for improving neurological features in neonatal diabetes: a systematic review and meta‐analyses. Pediatr Diabetes. 2022;23(6):675‐692. 35657808 10.1111/pedi.13376

[6] Beltrand J , Baptiste A , Busiah K , et al. Glibenclamide oral suspension: suitable and effective in patients with neonatal diabetes. Pediatr Diabetes. 2019;20(3):246‐254. 30684309 10.1111/pedi.12823

[7] Berdugo M , Delaunay K , Naud M , et al. The antidiabetic drug glibenclamide exerts direct retinal neuroprotection. Trans Res. 2021;229:83‐99. 10.1016/j.trsl.2020.10.003 33080394

[8] Richards S , Aziz N , Bale S , et al. Standards and guidelines for the interpretation of sequence variants: a joint consensus recommendation of the American College of Medical Genetics and Genomics and the Association for Molecular Pathology. Genet Med. 2015;17(5):405‐424. doi:10.1038/gim.2015.30 10.1038/gim.2015.30 PMC4544753 25741868

[9] De Franco E , Saint‐Martin C , Brusgaard K , et al. Update of variants identified in the pancreatic β‐cell K ATP channel genes KCNJ11 and ABCC8 in individuals with congenital hyperinsulinism and diabetes. Hum Mutat. 2020;41(5):884‐905. 32027066 10.1002/humu.23995 PMC7187370

[10] Galderisi A , Facchinetti A , Steil GM , et al. Continuous glucose monitoring in very preterm infants: a randomized controlled trial. Pediatrics. 2017;140(4):e20171162. doi:10.1542/peds.2017‐1162 28916591 10.1542/peds.2017-1162

[11] Beardsall K , Vanhaesebrouck S , Ogilvy‐Stuart AL , et al. Prevalence and determinants of hyperglycemia in very low birth weight infants: cohort analyses of the NIRTURE study. J Pediatr. 2010;157(5):715‐9.e1‐715‐9.e3. 20570286 10.1016/j.jpeds.2010.04.032

[12] Sabzehei M , Afjeh S , Shakiba M , Alizadeh P , Shamshiri A , Esmaili F . Hyperglycemia in VLBW infants; incidence, risk factors and outcome. Arch Iran Med. 2014;17(6):429‐434. 24916529

[13] Lemelman M , Letourneau L , Greeley S . Neonatal diabetes mellitus: an update on diagnosis and management. Clin Perinatol. 2018;45(1):41‐59. 29406006 10.1016/j.clp.2017.10.006 PMC5928785

[14] Beardsall K , Vanhaesebrouck S , Ogilvy‐Stuart AL , et al. A randomised controlled trial of early insulin therapy in very low birth weight infants, "NIRTURE" (neonatal insulin replacement therapy in Europe). BMC Pediatr. 2007;7:29. 17692117 10.1186/1471-2431-7-29 PMC1994677

[15] Shah R , McKinlay CJ , Harding JE . Neonatal hypoglycemia: continuous glucose monitoring. Curr Opin Pediatr. 2018;30(2):204‐208. 29346140 10.1097/MOP.0000000000000592 PMC5882205

[16] Galderisi A , Trevisanuto D , Russo C , Hall R , Bruschettini M . Continuous glucose monitoring for the prevention of morbidity and mortality in preterm infants. Cochrane Database Syst Rev. 2021;12(12):CD013309. doi:10.1002/14651858.CD013309 10.1002/14651858.CD013309.pub3 PMC8690212 34931697

[17] Quinn GEYG , Bell EF , Donohue PK , et al. Incidence and early course of retinopathy of prematurity: secondary analysis of the postnatal growth and retinopathy of prematurity (G‐ROP) study. JAMA Ophthalmology. 2022;136(12):1383‐1389. 10.1001/jamaophthalmol.2018.4290 PMC6583045 30326046

[18] Kermorvant‐Duchemin E , Le Meur G , Plaisant F , et al. Thresholds of glycemia, insulin therapy, and risk for severe retinopathy in premature infants: a cohort study. PLoS Med. 2020;17(12):e1003477. 33306685 10.1371/journal.pmed.1003477 PMC7732100

