# Correction to “Early treatment of neonatal diabetes with oral glibenclamide in an extremely preterm infant”

**DOI:** 10.1002/jmd2.12406

**Published:** 2023-12-26

Galderisi A, Kermorvant‐Duchemin E, Daruich A, et al. Early treatment of neonatal diabetes with oral glibenclamide in an extremely preterm infant. *JIMD Rep*. 2023;64(2):161–166. doi:10.1002/jmd2.12358

There is an error in the following sentence: “The two approved oral formulations of Amglidia include, besides glibenclamide, sodium benzoate (as a preservative), a lactic acid/sodium citrate mixture to give the solution a flavorless slight acid pH (= 4.80) that ensures the efficacy of sodium benzoate as preservative, and hydroxyethyl cellulose, and xanthan to prevent sedimentation of the active glibenclamide. Such a formulation has an osmolarity of **282 mOsm/L**, close to isotonicity, and can be administered either as a standalone suspension or diluted in milk.”

We would like to amend the published manuscript as follows: “The two approved oral formulations of Amglidia include, besides glibenclamide, sodium benzoate (as a preservative), a lactic acid/sodium citrate mixture to give the solution a flavorless slight acid pH (= 4.80) that ensures the efficacy of sodium benzoate as preservative, and hydroxyethyl cellulose, and xanthan to prevent sedimentation of the active glibenclamide. Such a formulation has an average osmolarity of **213 mOsm/L**, close to isotonicity, and can be administered either as a standalone suspension or diluted in milk.”

The authors apologize for the errors.

